# Combination of CBT and rTMS: what are the advantages?

**DOI:** 10.1192/j.eurpsy.2024.1478

**Published:** 2024-08-27

**Authors:** M. Kiss-Leizer, A. Á. Horváth, B. Kabella, L. Pogány, J. Lazáry

**Affiliations:** Psychiatry, National Institute of Menthal Health, Neurology and Neurosurgery - Nyírő Gyula Center, Budapest, Hungary

## Abstract

**Introduction:**

Obsessive-Compulsive Disorder (OCD) and Major Depressive Disorder (MDD) are among the ten most disabling disorders, yet only 30-40% of people with the condition seek specialist care (WHO). Considered a relatively new tool in the treatment of OCD and MDD, repetitive transcranial magnetic stimulation (rTMS) was first used by our team 1 year ago. Based on current literature cognitive behavioural therapy (CBT) is effective in 60% of OCD cases, with MDD also having a good response rate of 50-60%. The efficacy of SSRI’s has been demonstrated, but side effects can have a negative impact on adherence in the long term. Prolonged use of certain drugs has adverse reactions that lead specifically to memory impairment, which compromises suitability for psychotherapy. The same problem applies to the use of electroconvulsive therapy (ECT) in a major depressive episode.

**Objectives:**

Our aim was to study the efficacy of combining rTMS with CBT, to gather clinical experience on how these two different methods work in practice when combined.

**Methods:**

Patients diagnosed with therapy resistant MDD received rTMS treatment using 50Hz theta burst over 10 sessions. Therapy resistant OCD patients were treated by a 15 sessions rTMS using 1Hz single pulse. These rTMS sessions were combined with CBT, of which we would like to highlight two cases: one of them is a 34 years old woman, who has wide range of sexual, checking and contamination-related OCD symptoms and only received SSRI treatment so far. The other one is a 29 years old man, who suffers from religious obsessions, cleaning compulsions and other repetitive behaviours.

**Results:**

The positive effects of rTMS treatment on working memory functions, attentional capacity and cooperative skills without significant additive effects suggest exciting possibilities for combining the two treatments, thus the combined treatment has been tested in clinical practice. In our own patient care, an important experience was that patients were committed to the therapy, felt safe and, unlike with medications, did not have to worry about side effects. While medication and ECT can make psychotherapy more difficult in the long term - mainly because of memory problems - rTMS facilitates it. Patients appreciate that we approach their problems in a complex way, and they perceive that the combination of the two very different methods reflects professionalism. Our poster attempts to present the experience of combining rTMS and CBT from the therapist’s perspective through two case studies.

**Conclusions:**

Based on our experiences it is an effective approach to combine rTMS with CBT in therapy resistant MDD and OCD patients. In the light of these results the revision of the existing guidelines are considerable.

Acknowledgements

This study has been supported by the Human Resource Development Operating Programme 5.2.6 grant.

**Disclosure of Interest:**

None Declared

